# Norovirus Disease Among Children <5 Years in 3 Sub-Saharan African Countries: Findings From the Vaccine Impact on Diarrhea in Africa (VIDA) Study, 2015–2018

**DOI:** 10.1093/cid/ciac967

**Published:** 2023-04-19

**Authors:** Richard Omore, Helen Powell, Samba O Sow, M Jahangir Hossain, Billy Ogwel, Sanogo Doh, John B Ochieng, Joquina Chiquita M Jones, Syed M A Zaman, Alex O Awuor, Jane Juma, Irene N Kasumba, Anna Roose, Leslie P Jamka, Dilruba Nasrin, Jie Liu, Adama Mamby Keita, Awa Traoré, Uma Onwuchekwa, Henry Badji, Golam Sarwar, Martin Antonio, Ciara E Sugerman, Eric D Mintz, Eric R Houpt, Jennifer R Verani, Marc-Alain Widdowson, Sharon M Tennant, James A Platts-Mills, Jacqueline E Tate, Umesh D Parashar, Karen L Kotloff

**Affiliations:** Kenya Medical Research Institute, Center for Global Health Research (KEMRI-CGHR), Kisumu, Kenya; Department of Pediatrics, Center for Vaccine Development and Global Health, University of Maryland School of Medicine, Baltimore, Maryland, USA; Centre pour le Développement des Vaccins du Mali (CVD-Mali), Bamako, Mali; Centre pour le Développement des Vaccins du Mali (CVD-Mali), Bamako, Mali; Medical Research Council Unit, The Gambia at the London School of Hygiene & Tropical Medicine, Banjul, The Gambia; Kenya Medical Research Institute, Center for Global Health Research (KEMRI-CGHR), Kisumu, Kenya; Centre pour le Développement des Vaccins du Mali (CVD-Mali), Bamako, Mali; Kenya Medical Research Institute, Center for Global Health Research (KEMRI-CGHR), Kisumu, Kenya; Medical Research Council Unit, The Gambia at the London School of Hygiene & Tropical Medicine, Banjul, The Gambia; Medical Research Council Unit, The Gambia at the London School of Hygiene & Tropical Medicine, Banjul, The Gambia; Kenya Medical Research Institute, Center for Global Health Research (KEMRI-CGHR), Kisumu, Kenya; Kenya Medical Research Institute, Center for Global Health Research (KEMRI-CGHR), Kisumu, Kenya; Department of Medicine, Center for Vaccine Development and Global Health, University of Maryland School of Medicine, Baltimore, Maryland, USA; Department of Pediatrics, Center for Vaccine Development and Global Health, University of Maryland School of Medicine, Baltimore, Maryland, USA; Department of Medicine, Center for Vaccine Development and Global Health, University of Maryland School of Medicine, Baltimore, Maryland, USA; Department of Medicine, Center for Vaccine Development and Global Health, University of Maryland School of Medicine, Baltimore, Maryland, USA; Division of Infectious Diseases and International Health, Department of Medicine, University of Virginia, Charlottesville, Virginia, USA; Centre pour le Développement des Vaccins du Mali (CVD-Mali), Bamako, Mali; Centre pour le Développement des Vaccins du Mali (CVD-Mali), Bamako, Mali; Centre pour le Développement des Vaccins du Mali (CVD-Mali), Bamako, Mali; Medical Research Council Unit, The Gambia at the London School of Hygiene & Tropical Medicine, Banjul, The Gambia; Medical Research Council Unit, The Gambia at the London School of Hygiene & Tropical Medicine, Banjul, The Gambia; Medical Research Council Unit, The Gambia at the London School of Hygiene & Tropical Medicine, Banjul, The Gambia; Division of Foodborne, Waterborne, and Environmental Diseases, US Centers for Disease Control and Prevention, Atlanta, Georgia, USA; Division of Foodborne, Waterborne, and Environmental Diseases, US Centers for Disease Control and Prevention, Atlanta, Georgia, USA; Division of Infectious Diseases and International Health, Department of Medicine, University of Virginia, Charlottesville, Virginia, USA; Division of Global Health Protection, US Centers for Disease Control and Prevention, Nairobi, Kenya; Division of Global Health Protection, US Centers for Disease Control and Prevention, Nairobi, Kenya; Department of Medicine, Center for Vaccine Development and Global Health, University of Maryland School of Medicine, Baltimore, Maryland, USA; Division of Infectious Diseases and International Health, Department of Medicine, University of Virginia, Charlottesville, Virginia, USA; Division of Viral Diseases, US Centers for Disease Control and Prevention, Atlanta, Georgia, USA; Division of Viral Diseases, US Centers for Disease Control and Prevention, Atlanta, Georgia, USA; Department of Pediatrics, Center for Vaccine Development and Global Health, University of Maryland School of Medicine, Baltimore, Maryland, USA; Department of Medicine, Center for Vaccine Development and Global Health, University of Maryland School of Medicine, Baltimore, Maryland, USA

**Keywords:** diarrhea, norovirus, burden, children, Africa

## Abstract

**Background:**

To address a paucity of data from sub-Saharan Africa, we examined the prevalence, severity, and seasonality of norovirus genogroup II (NVII) among children <5 years old in The Gambia, Kenya, and Mali following rotavirus vaccine introduction.

**Methods:**

Population-based surveillance was conducted to capture medically-attended moderate-to-severe diarrhea (MSD) cases, defined as a child 0–59 months old passing ≥3 loose stools in a 24-hour period with ≥1 of the following: sunken eyes, poor skin turgor, dysentery, intravenous rehydration, or hospitalization within 7 days of diarrhea onset. Diarrhea-free matched controls randomly selected from a censused population were enrolled at home. Stools from cases and controls were tested for enteropathogens, including norovirus and rotavirus, by TaqMan quantitative polymerase chain reaction (PCR) and conventional reverse transcription PCR. We used multiple logistic regression to derive adjusted attributable fractions (AFe) for each pathogen causing MSD, which takes into consideration the prevalence in both cases and controls, for each site and age. A pathogen was considered etiologic if AFe was ≥0.5. In further analyses focusing on the predominant NVII strains, we compared rotavirus and NVII severity using a 20-point modified Vesikari score and examined seasonal fluctuations.

**Results:**

From May 2015 to July 2018, we enrolled 4840 MSD cases and 6213 controls. NVI was attributed to only 1 MSD episode. NVII was attributed to 185 (3.8%) of all MSD episodes and was the sole attributable pathogen in 139 (2.9%); peaking (36.0%) at age 6–8 months with majority (61.2%) aged 6–11 months. MSD cases whose episodes were attributed to NVII alone compared with rotavirus alone were younger (median age, 8 vs 12 months, *P* < .0001) and had less severe illness (median Vesikari severity score, 9 vs 11, *P* = .0003) but equally likely to be dehydrated. NVII occurred year-round at all study sites.

**Conclusions:**

Infants aged 6–11 months bear the greatest burden of norovirus disease, with NVII predominating. An early infant vaccine schedule and rigorous adherence to guidelines recommended for management of dehydrating diarrhea may offer substantial benefit in these African settings.

## Teaser

Norovirus genogroup II was the attributed etiology for 3% of all moderate-to-severe diarrhea (MSD) cases among children <5 years old in The Gambia, Mali, and Kenya post-rotavirus vaccine introduction; the highest disease burden concentrated in children 6–11 months old.

Although an increasing number of viruses that have been associated with diarrheal disease in humans during the past 4 decades [1], rotavirus and norovirus remain the 2 most common globally [2–4], particularly before rotavirus vaccine (RVV) introduction [5]. Although rotavirus prevalence diminished post-RVV introduction in a number of developed settings [6–10], limited data from sub-Saharan Africa suggest it remains a common pathogen in this region. In some developed countries where RVV has been introduced, norovirus is emerging as the most prevalent enteric virus [1, 4, 7–12]. As RVVs are introduced in sub-Saharan African countries, it is important to understand the relative contribution of norovirus and whether norovirus will subsequently fill the environmental niche of rotavirus in these settings. Norovirus epidemiology, circulating strains, peak age groups, clinical signs, and seasonality can guide vaccine development and intervention strategies [13]. Although such data are readily available for developed countries [13], similar data are sparse and remain insufficient to guide interventions in sub-Saharan Africa [14]. Computational simulation models suggest that the burden in Africa is large, with children <5 years old accounting for 15% (approximately 70 million) of the annual global norovirus cases and an estimated 40% (∼38 000) of annual global norovirus deaths [15].

The Global Enteric Multicenter Study (GEMS) elucidated the incidence, etiology, and adverse clinical consequences of moderate-to-severe diarrhea (MSD) among children under 5 years of age living in censused populations in low- and middle-income countries (LMIC) [16, 17]. As a follow-up to GEMS, the Vaccine Impact on Diarrhea in Africa (VIDA) study was conducted at 3 of the GEMS sites located in sub-Saharan Africa (The Gambia, Mali, and Kenya) after RVV introduction [18]. This article is nested in VIDA and aims to estimate the burden and describe prevalence, severity, and seasonality of norovirus among children <5 years residing in these 3 sub-Saharan African countries.

## METHODS

The study design, clinical and epidemiological procedures, and laboratory methods for VIDA are largely the same as those described previously for GEMS [16, 19] with updates related to VIDA described by Powell et al [20].

### Study Design and Participants

Leveraging health and demographic surveillance systems (DSS) located in the 3 sub-Saharan African countries, we maintained an updated censused population to provide a sampling frame for the case-control study. MSD cases were enrolled from sentinel health centers (SHCs) serving the DSS populations. Children were enrolled in 3 age strata (infants [0–11 months], toddlers [12–23 months], and young children [24–59 months]) according to the following inclusion criteria: diarrhea (≥3 looser than normal stools within 24 hours) with onset within the past 7 days, and ≥1 of the following: sunken eyes (more than normal), decreased skin turgor, dysentery (bloody stool), intravenous rehydration, or hospitalization. Episodes lasting >7 days were excluded. For every enrolled MSD case, we enrolled 1–3 diarrhea-free community controls at home. Controls were randomly selected from DSS database matched to cases by sex, age, and geography and were enrolled within 14 days of the case. At enrollment, study staff interviewed caretakers for each case and control to solicit demographic, epidemiological, and illness history and performed a focused physical examination; all participants provided a fresh stool sample.

### Laboratory Procedures

Stools collected from all cases and the first matched control were tested for a panel of enteropathogens, including rotavirus and norovirus, by TaqMan^®^ Array Card (TAC) in which quantitative polymerase chain reaction (qPCR) cycle threshold (Ct) cutoff values <35 were considered qPCR positive for the pathogen detected as described elsewhere by Liu et al [21]. Stools from all cases and controls were also tested for norovirus by conventional reverse-transcription polymerase chain reaction (RT-PCR) as described previously [19].

### Statistical Analysis

The statistical methods used in VIDA were consistent with those used in GEMS and have been described previously [16] and are further elaborated by Powell et al [20]. Specific statistical details relevant to the current norovirus analysis are summarized below.

### Etiologic (Attributable) Detection of Norovirus GI (NVI) and GII (NVII)

The adjusted odds ratio for each individual MSD case was estimated using a conditional logistic regression model [20] that includes the pathogen, an interaction between pathogen and site and between pathogen and age group, as well as all other pathogens that occurred in at least 2% of cases and controls. Using the adjusted model coefficients, we estimated the individual's episode-specific odds ratio (ORe), which was converted to the episode-specific attributable fraction (AFe) using AFe=1−(1ORe). Cases with AFe ≥ 0.5 were considered etiologic for the individual pathogen as previously described [20, 22, 23]. AFe ≥ 0.5 translates to a pathogen-specific cycle threshold (Ct) value associated with a 2-fold higher likelihood of being an MSD case (vs a control) and was considered “attributed,” that is, achieving criteria for having a likely causal association with the MSD episode.

### Comparing Attributable Cases

After finding a low attributable burden of norovirus genogroup I (NVI), we limited additional analyses of attributable cases to children infected with NVII. We assessed the age and season distribution of norovirus genogroup II (NVII) and compared the clinical features and severity of MSD episodes attributable to NVII to those attributable to rotavirus excluding MSD episodes with other possible co-etiologies. We examined the proportion of MSD cases in which NVII was the sole attributable fraction versus a coinfection with any 1 of the more than 20 potential enteropathogens identifiable by the TAC qPCR. Continuous variables were compared using a Wilcoxon rank sum test and dichotomous/binary and categorical variables were compared using χ^2^ or Fisher exact test, as appropriate. Severity was measured by duration of diarrhea, maximum number of watery diarrhea episodes in a 24-hour period, vomiting, fever, dehydration [24], and treatment recommendation using a 20-point modified Vesikari score [24]. A *P* value <.05 was considered statistically significant.

We obtained weather data from the following: Government of The Gambia Annual Climate Report 2018 (The Gambia); Africa climate data site https://en.climate-data.org/africa/ [Accessed 20 September 2019] (Mali), and the Kenya Meteorological Department, Kisumu International Airport station (Kenya). We used an average temperature (degrees Celsius) based on the maximum and minimum temperature for each month, which was averaged over the study period. We also used monthly rainfall (millimeters) averaged over the study period.

### Ethical Review

This analysis involves data collected as part of the VIDA protocol, which was reviewed and approved by the Institutional Review Board (IRB) of the University of Maryland, Baltimore, Maryland, USA (HP-00062472), the Centers for Disease Control and Prevention (CDC) (reliance agreement 6729), The Gambia Government/Medical Research Council/Gambia at the London School of Hygiene & Tropical Medicine (1409), the Comité d'Ethique de la Faculté de Médecine, de Pharmacie, et d'Odonto-Stomatologie, Bamako, Mali (no number), and the Kenya Medical Research Institute Scientific & Ethics Review Unit in Siaya County, Kenya (SSE 2996). Written informed consent was obtained from the parent(s) or primary caretaker (s) of each child who met eligibility criteria before any research activities were performed.

## RESULTS

### Norovirus Prevalence by Site and Overall

A total of 4840 MSD cases and 6213 matched diarrhea-free control children were enrolled. The site-specific distribution of MSD cases and controls and the prevalence of norovirus by RT-PCR and qPCR are shown in Supplementary Figure 1. qPCR detected a higher percentage of norovirus compared to conventional RT-PCR at all sites (Table 1). The overall prevalence of either NVI or NVII by qPCR among VIDA cases was (776/4806 [16.1%]), which was similar to that of controls (749/4775 [15.7%]). An additional 28 cases and 20 controls had mixed NVI and NVII infections (Table 1). Only 1 (<0.1%) MSD case who tested by qPCR was considered to be attributable to NVI and 185 (3.8%) to NVII, which represents <0.1% and 33.4% of the qPCR positive cases, respectively. We excluded 46 of the 189 attributable NVII cases from further analysis because they were attributed to NVII in addition to at least 1 other enteric pathogen, mainly enterotoxigenic *Escherichia coli* encoding heat-stable enterotoxin (ST-ETEC) (n = 12), *Helicobacter pylori* (n = 11), *Cryptosporidium* (n = 8), rotavirus (n = 5), or *Shigella* (n = 5). In total, 139 (2.9%) of the 4807 MSD cases were included in the final analysis as they were attributable to NVII as a single agent. Regardless of study site and age group, 2779 (57.4%) of the 4840 MSD cases, tested by TAC qPCR, could be attributed to at least 1 enteric pathogen.

**Table 1.** Proportion of Children Who Were Positive for Norovirus by Conventional RT-PCR or TAC qPCR (Where Positivity Is Defined as a Pathogen-Specific Cycle Threshold (Ct) < 35), and the Proportion Who Had an Attributable Detection of Norovirus (AFe ≥ 0.5) by qPCR in the VIDA Study, 2015–2018

	NVI		NVII							
	All Sites		All Sites		The Gambia		Mali		Kenya	
Enrolled	Cases (N = 4840)	Controls (N = 6213)	Cases (N = 4840)	Controls (N = 6213)	Cases (N = 1678)	Controls (N = 2138)	Cases (N = 1608)	Controls (N = 1980)	Cases (N = 1554)	Controls (N = 2095)
No. (%) positive by RT-PCR^a^	66/4838 (1.4%)	97/6209 (1.6%)	218/4838 (4.5%)	219/6209 (3.5%)	77/1678 (4.6%)	91/2138 (4.3%)	46/1607 (2.9%)	51/1979 (2.6%)	95/1553 (6.1%)	77/2092 (3.7%)
No. (%) positive (Ct < 35) by qPCR^a^	224/4806 (4.7%)	233/4775 (4.9%)	553/4807 (11.5%)	516/4775 (10.8%)	178/1655 (10.8%)	179/1643 (10.9%)	177/1604 (11.0%)	185/1601 (11.6%)	198/1548 (12.8%)	152/1531 (9.9%)
No. (%) attributable (AFe ≥ 0.5)	1/4806 (<0.1%)	…	185/4807 (3.8%)	…	38/1655 (2.3%)	…	45/1604 (2.8%)	…	102/1548 (6.6%)	…

Abbreviations: Afe, attributable fraction; NV, norovirus; NVI, norovirus genogroup I; NVII, norovirus genogroup II; qPCR, quantitative polymerase chain reaction; RT-PCR, reverse-transcription polymerase chain reaction; TAC, TaqMan^®^ Array Card; VIDA, Vaccine Impact on Diarrhea in Africa.

All cases and controls were tested by RT-PCR, whereas all cases but only the first matched control were tested by qPCR. Note that 28 cases and 20 controls had both NVI and NVII detected by qPCR, and 2 controls had both NVI and NVII detected by RT-PCR. The denominators are the total number for whom the laboratory test was successfully conducted.

### Age Distribution and Seasonality of NVII Etiologic Cases

**Figure 1.**
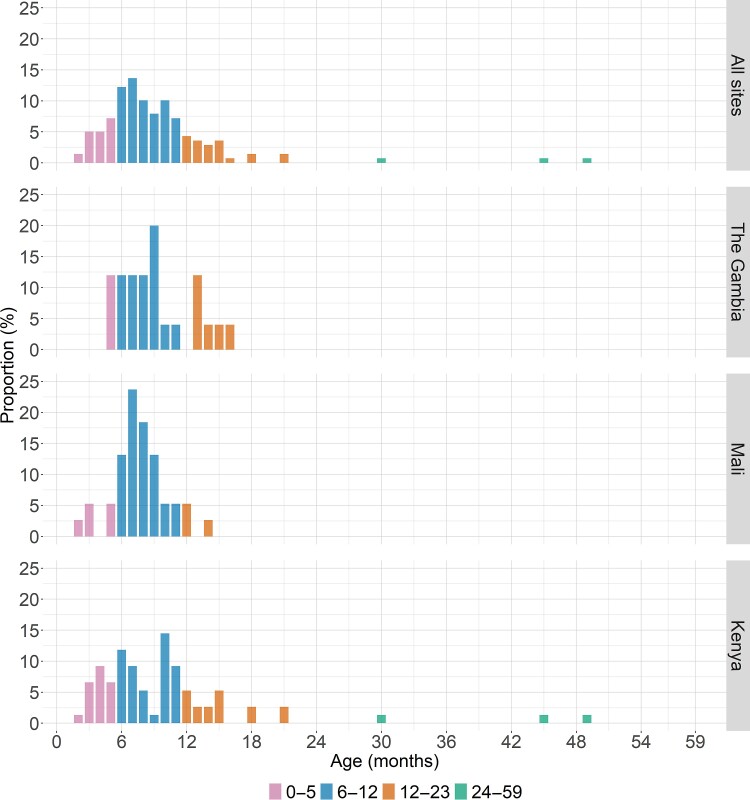
Frequency distribution of 139 cases of moderate-to-severe diarrhea where NVII was the single attributed pathogen (AFe ≥ 0.5) by age group and study site in the Vaccine Impact on Diarrhea in Africa (VIDA) study (2015–2018). Abbreviations: Afe, attributable fraction; NVII, norovirus genogroup II.

Regardless of study site, the 139 cases with NVII as the single attributable etiology of MSD were rarely observed among young infants aged 0–2 months (n = 2 [1.4%]) but were more frequently observed among infants aged 3–5 months (n = 24 [17.3%]), peaking in the second half of infancy, 6–8 months (n = 50 [36.0%]) (Figure 1). The number of cases declined among infants aged 9–11 months (n = 35 [25.2%], toddlers 12–23 months (n = 25 [18.0%]), and young children aged 24–59 months (n = 3 [2.2%]). In examining these age groups, the majority (85/139 [61.2%] of NVII single etiologic cases in this study were aged 6–11 months and 110/139 (79.1%) were aged 6–23 months. NVII was the sole pathogen identified in most of the MSD episodes in which NVII met criteria for being attributable at all three sites (Supplementary Figures 2*A* and 2*B* ). This trend was most apparent in the 6–11 month age group when most NVII infections occurred.

**Figure 2.**
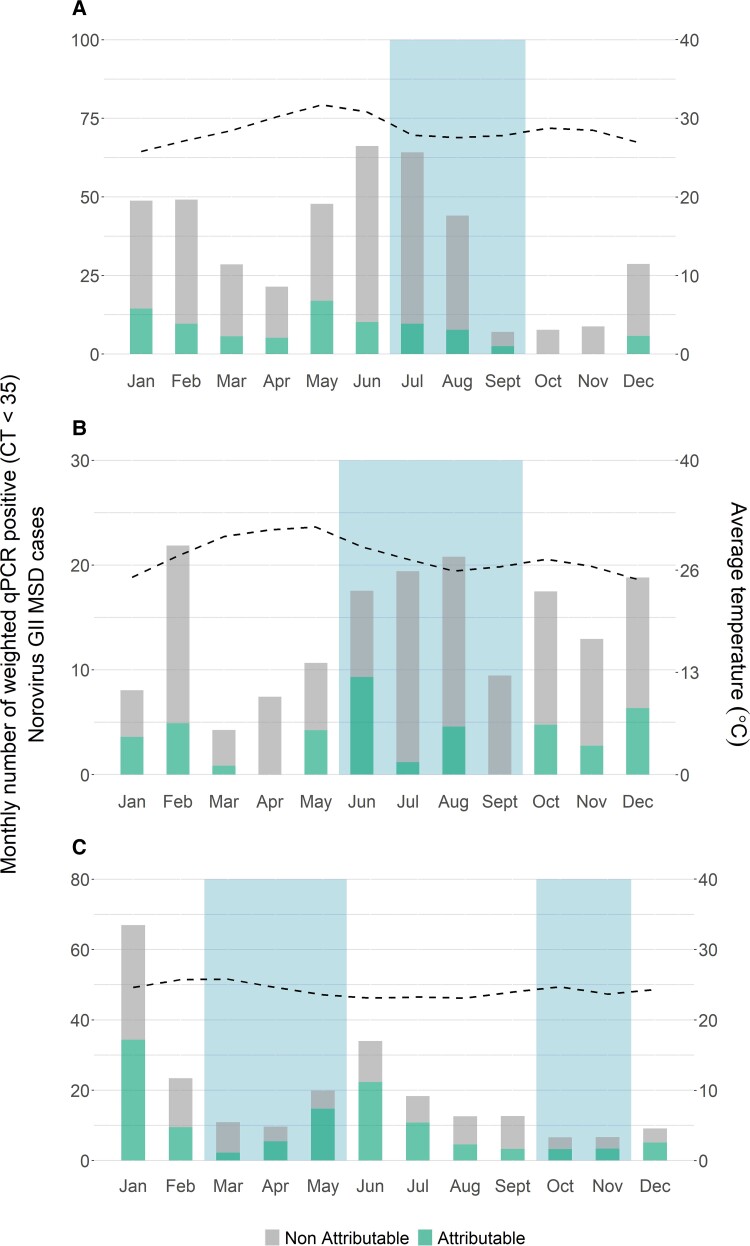
Seasonality of NVII attributable and non-attributable MSD cases in: (*A*) The Gambia, (*B*) Mali and (*C*) Kenya, in the Vaccine Impact on Diarrhea in Africa (VIDA) study (2015–2018). Blue bars represent the rainy season(s), and the dotted line represents the average temperature. Abbreviations: MSD, moderate-to-severe diarrhea; NVII, norovirus genogroup II.

NVII detection occurred year-round at different levels at each site during our study period (Figure 2). Distinct seasonal patterns by rainfall, month, or temperature were not apparent for either any PCR positive NVII or for NVII as an attributable etiology.

### Characteristics of MSD Episodes With Either NVII or Rotavirus as the Single Attributable Etiology

We examined and compared the clinical features of 139 MSD episodes with NVII as the single attributable etiology to 483 MSD episodes with rotavirus as the single attributable etiology. At enrollment, a similar proportion (*P* > .05 for all) of MSD cases with NVII and rotavirus had blood in stool observed by the parent, clinician, or lab staff (4.3% vs 5.4%), fever >38.4°C axillary (3.6% vs 5.6%) and malnutrition, defined as mid-upper arm circumference <12.5 (20.1% vs 15.5%, respectively). Compared to MSD cases with rotavirus, those with NVII were younger (median age: 8 months (interquartile range [Q1–Q3]: 6–11 vs 12 months [Q1–Q3: 8–19], *P* < .0001) and more likely to be male (83/139 [59.7%] vs 258/483 [53.4%], although the latter comparison did not reach statistical significance (*P* = .2234).

Using the criteria present in the modified Vesikari score (Supplementary Table 1), MSD attributable to rotavirus alone appeared more severe than MSD attributable to NVII alone based on a significantly higher median score (11 [Q1–Q3: 8–12] vs 9 [Q1–Q3: 7–11], *P* = .0003) and a greater proportion of episodes in the severe category (246/483 [50.9%] vs 42/139 [30.2%], *P* < .0001) (Table 2). In particular, those with rotavirus were significantly more likely to vomit (378/483 [78.3%] vs 93/139 [66.9%], *P* = .0083) and to require intravenous fluid or hospitalization (75/483 [15.5%] vs 6/139 [4.3%], *P* = .0001). Nearly all children with either pathogen received either oral or IV rehydration.

**Table 2.** **Measures of Disease Severity** ^**a**^ **Among NVII Versus Rotavirus Cases of MSD Attributed to (AFe ≥ 0.5) With NVII or Rotavirus as a Single Etiology Among Children in The Gambia, Mali, and Kenya Participating in VIDA (2015–2018)**

Characteristics		NVII (N = 139)	Rotavirus (N = 483)	*P* ^b^
Duration of diarrhea (days)	Median (Q1–Q3)	3 (2, 4)	3 (2, 3)	.2182
Max no. loose stools in 24 hours	1–3	29 (20.9%)	77 (15.9%)	.4671
	4–5	85 (61.2%)	301 (62.3%)	
	6–10	25 (18.0%)	103 (21.3%)	
	>10	0	2 (0.4%)	
Experienced vomiting	Yes	93 (66.9%)	378 (78.3%)	**.0083**
Max no. vomiting episodes on worst day^a^	1	13 (14.0%)	36 (9.5%)	.3479
	2–4	65 (69.9%)	265 (70.1%)	
	≥5	15 (16.1%)	77 (20.4%)	
Duration of vomiting (days)^c^	Median (Q1–Q3)	2 (1, 2)	2 (2, 3)	**.0216**
Axillary temperature (°C)	37.2–38.4	41 (29.5%)	170 (35.2%)	.4364
	38.5–38.9	3 (2.2%)	18 (3.7%)	
	>39	2 (1.4%)	9 (1.9%)	
	Median (Q1–Q3)	36.7 (36.4, 37.3)	36.9 (36.5, 37.5)	**.0147**
Dehydration (%)	No dehydration	5 (3.6%)	29 (6.0%)	.4053
	Some dehydration	109 (78.4%)	382 (79.1%)	
	Severe dehydration	25 (18.0%)	72 (14.9%)	
Treatment	None	0 (0.0%)	10 (2.1%)	**.0001**
	Oral rehydration only	133 (95.7%)	398 (82.4%)	
	IV rehydration or hospitalization	6 (4.3%)	75 (15.5%)	
mVS (integer)	Median (Q1–Q3)	9 (7, 11)	11 (8, 12)	**.0003**
mVS severity category	Mild	24 (17.3%)	65 (13.5%)	**<.0001**
	Moderate	73 (52.5%)	172 (35.6%)	
	Severe	42 (30.2%)	246 (50.9%)	

Abbreviations: Afe, attributable fraction; MSD, moderate-to-severe diarrhea; NV, norovirus; NVI, norovirus genogroup I; NVII, norovirus genogroup II; VIDA, Vaccine Impact on Diarrhea in Africa.

Includes parameters that comprise the modified Vesikari Score (mVS).

Two-tailed *P* values generated by χ^2^ test for categorical variables (except when the expected count was less than 5 in which case a Fisher exact test was used) and a Wilcoxon rank sum test for continuous variables. Statistically significant *P* values are shown in bold.

Among children with vomiting.

## DISCUSSION

Using a highly sensitive molecular assay, controlling for age, site, and other pathogens, we investigated the site-specific prevalence of norovirus as a cause of medically attended MSD among infants and young children following RVV introduction at three sites in sub-Saharan Africa, where data are sparse. We provided precise, site-specific prevalence estimates by adjusting for age, site, and other pathogens as well as asymptomatic carriage rates and viral load and characterized the seasonality and clinical manifestations among cases in which NVII was the sole attributable pathogen. We demonstrated that NVII is an important pathogen among infants seeking medical care with MSD in sub-Saharan Africa. Our findings corroborate and extend observations by others regarding the predominance of the NVII genogroup in Africa [25], and that circa 70% of pediatric norovirus diarrhea cases occur between age 6 and 23 months [26], commonly peaking between 6 and 11 months [11].

The high prevalence of norovirus among cases and controls 0–11 months of age in our study and elsewhere [27] suggests their potential role as reservoirs for norovirus transmission in the community. In addition, prolonged asymptomatic carriage in the face of immune pressure could promote emergence of antigenic variants [28] in this age group given the propensity of the norovirus genome to undergo mutations and recombination events [29]. The burden of norovirus in infants and their potential importance in disease transmission has propelled efforts to develop vaccines [30]. A review of the age distribution norovirus cases globally concluded that vaccinating prior to the peak age of infection at 6 months has the potential to prevent 85% of pediatric cases [26]; there is also a possibility that herd immunity engendered by broad uptake of a vaccine for infants could protect other age groups.

Consistent with previous studies [5, 21, 22], the TAC qPCR used in our study was more sensitive than RT-PCR in detecting norovirus. However, more infections were detected in both cases and controls; as a result, the proportion of MSD that could be attributed to norovirus did not increase significantly [21]. Similarly, high rates of asymptomatic infection also have been observed in birth cohort studies [31], resulting in attributable fractions of norovirus (∼5%–6%) that were 4-fold lower than the prevalence in children with diarrhea [11, 23]. We also corroborated observations from birth cohort studies that the prevalence of norovirus in both diarrheal and non-diarrheal stools varies widely across geographic locations [27]. In MAL-ED, the prevalence in diarrhea cases across sites ranged from 7.1% to 32.8% and in asymptomatic children from 2.2% to 30.4%.

Several factors may account for the high prevalence of asymptomatic infection in LMIC. One is that frequent exposure to norovirus leads to partial immunity that manifests as asymptomatic infection. Given evidence that homotypic immunity occurs, repeated exposure to the predominant pandemic GII.4 genotype could potentially serve as a natural immunization [32]. In contrast, others argue that immunity induced by a previous infection is unlikely to prevent illness in the large number of asymptomatic infants observed in LMIC [32]. A second hypothesis is that asymptomatic infections represent prolonged virus shedding from a recent symptomatic illness [33]. Prolonged shedding has been observed in immunocompetent and immunodeficient infants and adults [34–37]. Whereas case-control studies such as VIDA lacked the ability to account for prior infections, longitudinal birth cohort studies generally have not reported asymptomatic infections following norovirus diarrhea [11]. Moreover, 70% of the asymptomatic infections in the Ecuador birth cohort had no preceding diarrheal symptoms for at least 14 days [31]. A third hypothesis is that the use of highly sensitive qPCR detects low viral loads associated with subclinical infection. For this reason, we utilized the AFe to identify attributable infections based on higher viral loads that were associated with MSD. A fourth hypothesis is that the more virulent NVII genotypes such as the GII.4 strain might have predominated in cases while the asymptomatic infections comprised less pathogenic genotypes. Observations from a birth cohort in Ecuador seems to refute this hypothesis since the genotype distribution among diarrheal stools and healthy stools were similar [31]. A fifth hypothesis is that host genetic factors present on epithelial cell surfaces and in mucosal secretions, such as human histo-blood group antigens (HBGAs), might affect levels of asymptomatic infection. These cellular receptors for attachment of various norovirus genotypes [38, 39] are highly polymorphic, so genotype-specific susceptibility to norovirus may vary across populations. However, most studies suggest that both symptomatic and asymptomatic norovirus infection follow the same HBGA susceptibility patterns, with non-secretors resistant to both symptomatic and asymptomatic infections as a result of secretor-specific genotypes [40].

In VIDA we were also able to assess the population-based disease burden caused by NVII by calculating the population attributable fractions, as reported elsewhere [18]. We found that NVII was significantly associated with MSD among infants 0–11 months of age at each site with attributable fractions of 5.0%, 6.2%, and 10.8% in The Gambia, Mali and Kenya, respectively. Accordingly, NVII ranked as the 5th, 3rd, and 2nd most common pathogen significantly associated with MSD at these sites. However, among children aged 12–23 months, a significant association was seen only in Kenya with an attributable fraction of 5.9%. The reduced attributable fraction of NVII in Kenya during the second year of life compared to the first, and the lack of association with MSD at the other 2 sites in this age group, suggests that NVII may be causing less diarrhea in older children who have gained natural immunity from an initial infection acquired before one year of age. This hypothesis is supported by observations from the MAL-ED birth cohort study that after an initial NVII infection, children had a 27% reduction in risk of subsequent infection [11].

Our findings suggest that NVII could be in circulation year-round with intermittent peaks driven by factors that remain undetermined. These results corroborate other studies suggesting that a seasonal pattern is not apparent in more tropical regions including countries in Africa, whereas norovirus cases and outbreaks in temperate climates tend to show a clear seasonality with peaks in the winter months [33, 41]. As global warming increases and climate becomes more unpredictable, further understanding of the factors that drive peaks of norovirus transmission would be useful to inform control efforts.

Using the modified Vesikari score as a metric, we found that children with NVII as the sole attributed pathogen had significantly less severe illness compared to those with rotavirus as the sole attributed pathogen. In particular, NVII episodes had less vomiting and were less likely to require intravenous fluids or hospitalization. That most norovirus causes results in mild acute gastroenteritis has been observed in a systemic review and metanalysis of global norovirus infections [41]. Therefore, it can be expected that medical facility surveillance studies from sub-Saharan Africa such as VIDA may report fewer cases than community-based studies [42]. Nonetheless, the ability of NVII to cause severe dehydration should not be minimized.

Our current findings may need to be interpreted with caution until there is a more definitive understanding of the clinical significance of asymptomatic norovirus infection. If indeed asymptomatic infections are the consequence of recent symptomatic infections, then our estimates of NVII burden could underestimate the relative importance NVII in these settings. Although we attempted to control for confounders in our assessment of disease severity by limiting comparisons to MSD episodes that had high viral loads and excluding those with co-pathogens, there could be other confounders that we did not consider.

In summary, this study contributes to growing evidence in the literature showing NVII has a high infection burden among infants in sub-Saharan Africa and serves as an important cause of MSD among children seeking medical attention. The propensity of the virus to undergo genetic variation and disseminate globally poses a threat for more virulent strains to emerge. Development of a safe and effective vaccine for young infants, implemented concurrently with complementary interventions such as use of oral rehydration therapy, could offer substantial benefits.

## Supplementary Data

Supplementary materials are available at *Clinical Infectious Diseases* online. Consisting of data provided by the authors to benefit the reader, the posted materials are not copyedited and are the sole responsibility of the authors, so questions or comments should be addressed to the corresponding author.

## Supplementary Material

ciac967_Supplementary_Data

## Notes

***Acknowledgments.*** The authors wish to express our deep gratitude to the families who participated in these studies, the clinical and field staff for their exceptional hard work and dedication, and to the physicians, administration, and health officials at every site who generously provided facilities and support for the conduct of the study. They are grateful to Catherine Johnson, Chris Focht, and Nora Watson at the Emmes Company, LLC, for expert data management and reporting. Special thanks go to Carl Kirkwood, Duncan Steele, and Anita Zaidi at the Bill & Melinda Gates Foundation (BMGF) for helpful oversight, Kathy Neuzil for thoughtful suggestions, and to the following members of our International Scientific Advisory Committee for providing insightful comments and guidance: Janet Wittes (Chair), George Armah, John Clemens, Christopher Duggan, Stephane Helleringer, Ali Mokdad, James Nataro, and Halvor Sommerfelt. This supplement is sponsored by the Center for Vaccine Development and Global Health (CVD) at the University of Maryland School of Medicine, Baltimore (UMB).

***Supplement sponsorship.*** This supplement was sponsored by the Bill & Melinda Gates Foundation. This study is based on research funded in part by grants from the Bill & Melinda Gates Foundation (OPP1111236/OPP1116751).

***Ethical approval.*** This project was approved by the Institutional Review Boards (IRB) of the University of Maryland, Baltimore, MD, USA (HP-00062472), the US Centers for Disease Control and Prevention, Atlanta, GA, USA (reliance agreement, CDC protocol #: 6729), the Gambia Government/Medical Research Council/Gambia at the London School of Hygiene & Tropical Medicine (1409), the Comité d'Ethique de la Faculté de Médecine, de Pharmacie, et d'Odonto-Stomatologie, Bamako, Mali (no number), and the Kenya Medical Research Institute Scientific & Ethics Review Unit in Siaya County, Kenya (SSE 2996). Written, informed consent was obtained from the parent or primary caretaker of each child who met eligibility criteria before any research activities were performed.

***Financial support*.** This study was funded by the BMGF (grant numbers OPP1111236 and OPP1116751).
