# Supplementary material for: Norovirus Disease Among Children <5 Years in 3 Sub-Saharan African Countries: Findings From the Vaccine Impact on Diarrhea in Africa (VIDA) Study, 2015–2018
Source: Clin Infect Dis. 2023 Apr 19;76(Suppl 1):S114–22. doi: 10.1093/cid/ciac967 (PMC10116553; doi:10.1093/cid/ciac967)
Supplement: ciac967_Supplementary_Data [file ciac967_supplementary_data.zip › CID-116052-Omore_Norovirus_Supplemental_Clean.docx]

**SUPPLEMENTAL MATERIAL**

#

# **Norovirus disease among children <5 years in three sub-Saharan African countries: Findings from the Vaccine Impact on Diarrhea in Africa (VIDA) study, 2015-2018**

Richard Omore, Helen Powell, Samba O. Sow, M. Jahangir Hossain, Billy Ogwel, Sanogo Doh, John B. Ochieng, Joquina Chiquita M. Jones, Syed M.A. Zaman, Alex O. Awuor, Jane Juma, Irene N. Kasumba, Anna Roose, Leslie P. Jamka, Dilruba Nasrin, Jie Liu, Adama Mamby Keita, Awa Traoré, Uma Onwuchekwa, Henry Badji, Golam Sarwar, Martin Antonio, Ciara E. Sugerman, Eric D. Mintz, Eric R. Houpt, Jennifer R. Verani, Marc-Alain Widdowson, Sharon M. Tennant, James A. Platts-Mills, Jacqueline E.Tate, Umesh D. Parashar, and Karen L. Kotloff

**Supplemental Figure 1.** Proportion of MSD cases and diarrhea-free controls positive for norovirus by conventional RT-PCR and TAC qPCR, by site.

**Supplementary Figure 2.** Number (**Figure 2a**) and proportion (**Figure 2b**) of episodes of moderate-to-severe diarrhea (MSD) meeting criteria for being attributable to norovirus genotype II infection in which NVII was the sole attributable pathogen or in which other pathogens were present by quantitative polymerase chain reaction (qPCR), stratified by age group and site. Co-infecting pathogens that were tested by qPCR include adenovirus serotype 40/41, *Aeromonas* spp., astrovirus, *Bacteroides fragilis*, *Campylobacter* spp., *Cryptosporidium* spp., Enterocytozoon bieneusi, Helicobacter pylori, norovirus genotype I, Plesiomonas spp., rotavirus, Salmonella spp., sapovirus, Shigella spp/enteroinvasive Escherichia coli, enteroaggregative E. coli, heat stable toxin-producing enterotoxigenic E. coli, heat labile toxin- producing enterotoxigenic E. coli, typical enteropathogenic E. coli, atypical enteropathogenic E. coli, Shiga toxin-producing typical E. coli.

**Supplemental Table 1.** Comparison of Vesikari Score and the modified Vesikari Score used in VIDA


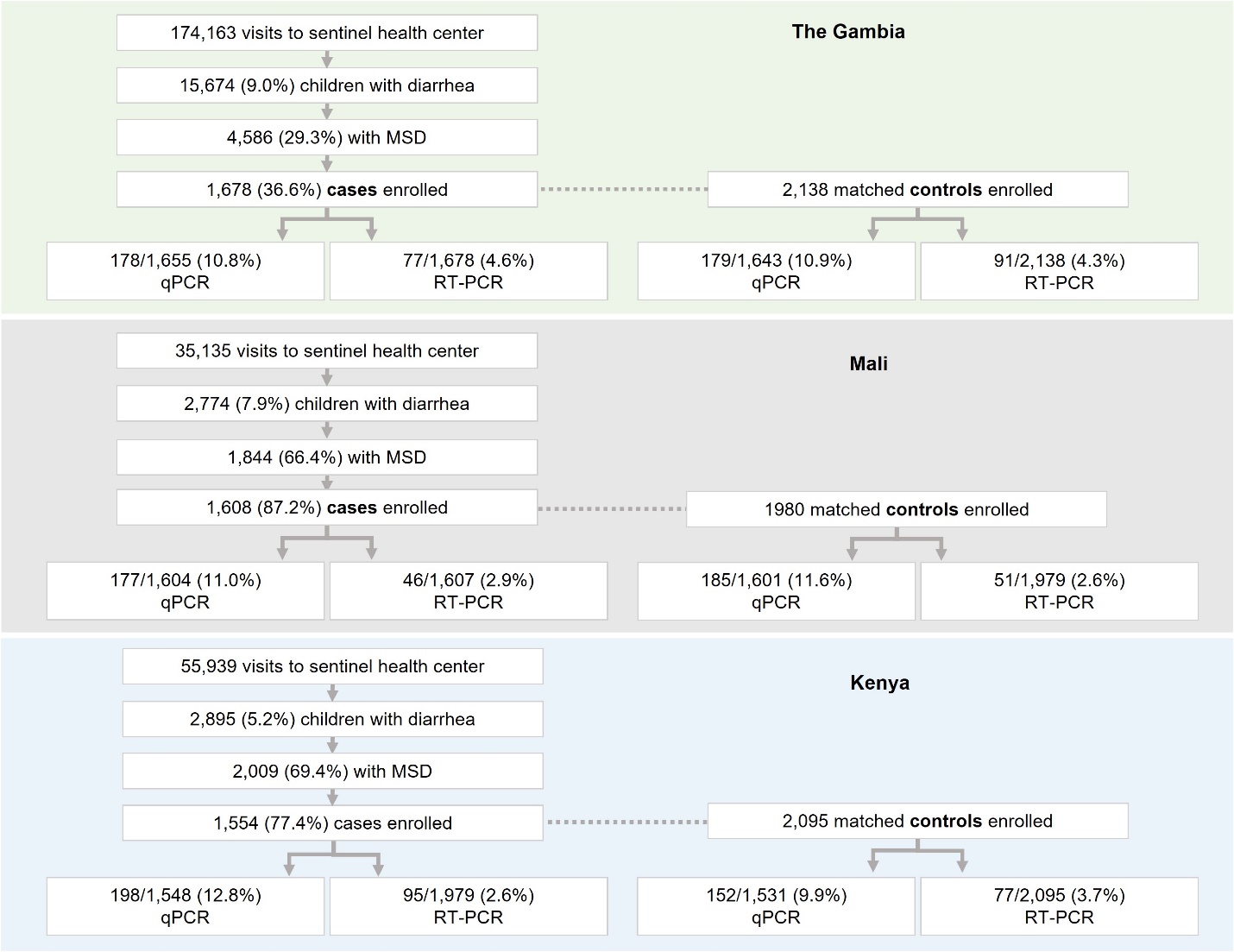


**Supplemental Figure 1. Proportion of MSD cases and diarrhea-free controls positive for norovirus by conventional RT-PCR and TAC qPCR, by site.**

a


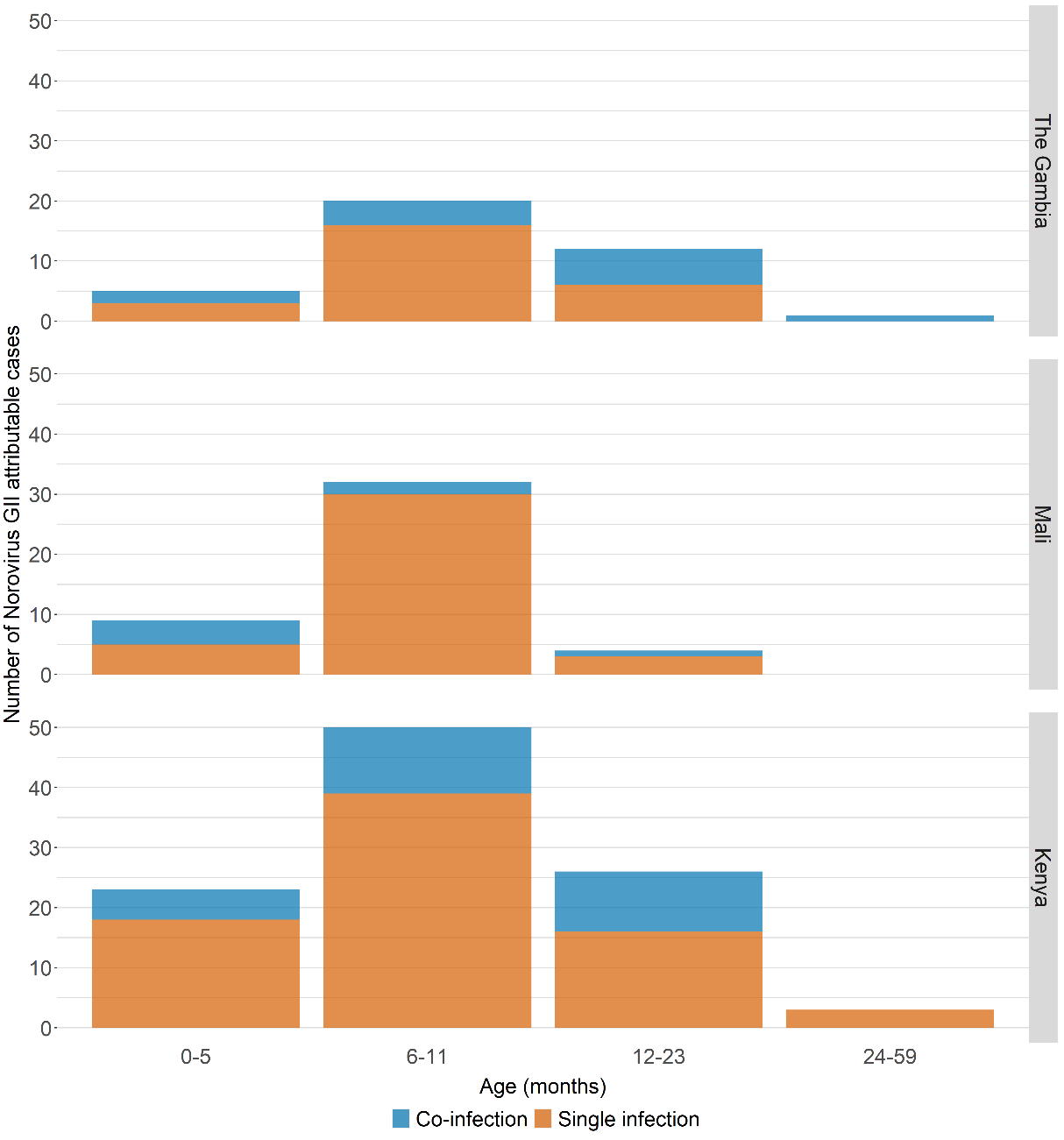


b


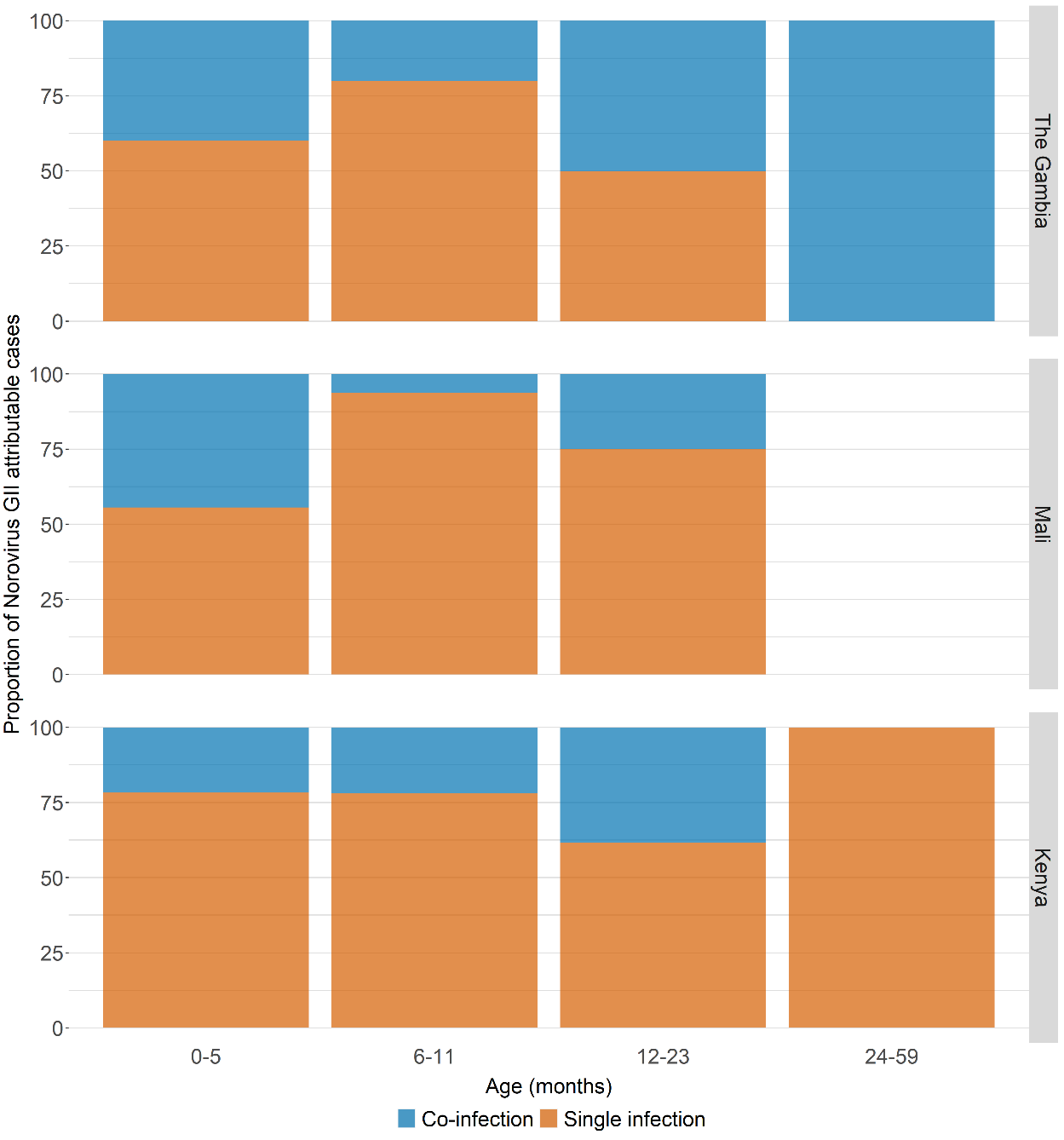


**Supplementary Figure 2.** Number (**Figure 2a**) and proportion (**Figure 2b**) of episodes of moderate-to-severe diarrhea (MSD) meeting criteria for being attributable to norovirus genotype II infection in which NVII was the sole attributable pathogen or in which other pathogens were present by quantitative polymerase chain reaction (qPCR). Co-infecting pathogens that were tested by qPCR include adenovirus serotype 40/41, *Aeromonas* spp., astrovirus, *Bacteroides fragilis*, *Campylobacter* spp., *Cryptosporidium* spp., *Enterocytozoon* bieneusi, Helicobacter pylori, norovirus genotype I, Plesiomonas spp., rotavirus, Salmonella spp., sapovirus, Shigella spp/enteroinvasive Escherichia coli, enteroaggregative E. coli, heat stable toxin-producing enterotoxigenic E. coli, heat labile toxin- producing enterotoxigenic E. coli, typical enteropathogenic E. coli, atypical enteropathogenic E. coli, Shiga toxin-producing typical E. coli.

| **Supplemental Table S1. Comparison of Vesikari Score and the modified Vesikari Score used in VIDA** | | | | | | |
| --- | --- | --- | --- | --- | --- | --- |
| **Parameter** | **Score** | | | | | **Comment** |
|  | **Vesikari** | | | | |  |
|  | **1** | **2** | | | **3** |  |
| Diarrhea |  |  | | |  |  |
| Max. no. stools/day | 1-3 | 4-5 | | | >6 |  |
| Duration (days) | 1-4 | 5 | | | >6 |  |
| Vomiting |  |  | | |  |  |
| Max. no. emesis/day | 1 | 2-4 | | | >5 |  |
| Duration (days) | 1 | 2 | | | >3 |  |
| Temperature (°C) | 37.1-38.4 | 38.5-38.9 | | | >39.0 |  |
| Dehydration | N/A | 1-5% or some | | | >6% or severe | . |
| Treatment | Rehydration | Hospitalization | | | N/A | Participants who are “hospitalized” for at least 24 hours OR who receive IV therapy are considered “hospitalized” and receive a corresponding score of 2 points for this parameter |
|  | **VIDA Modified Vesikari Score (mVS)** | | | | |  |
| Diarrhea |  | |  |  | |  |
| Max. no. stools/day | 3 | | 4-5 | >6 | | 1-2 days not permissible; definition of diarrhea requires >3 stools/day |
| Duration (days) | 1-4 | | 5 | 6-7 | | Cannot exceed 7; enrolment criteria requires <7 days |
| Vomiting |  | |  |  | |  |
| Max. no. emesis/day | 1 | | 2-4 | >5 | |  |
| Duration (days) | 1 | | 2 | >3 | | May be truncated because of diarrhea duration enrolment criterion |
| Temperature (°C) | 37.1-38.4 | | 38.5-38.9 | >39.0 | | No change |
| Dehydration | N/A | | some | severe | | No change |
| Treatment | Rehydration | | Hospitalization/IV | N/A | | No change |
