# Supplementary figures and images for: Norovirus Disease Among Children <5 Years in 3 Sub-Saharan African Countries: Findings From the Vaccine Impact on Diarrhea in Africa (VIDA) Study, 2015–2018
Source: Clin Infect Dis. 2023 Apr 19;76(Suppl 1):S114–22. doi: 10.1093/cid/ciac967 (PMC10116553; doi:10.1093/cid/ciac967)

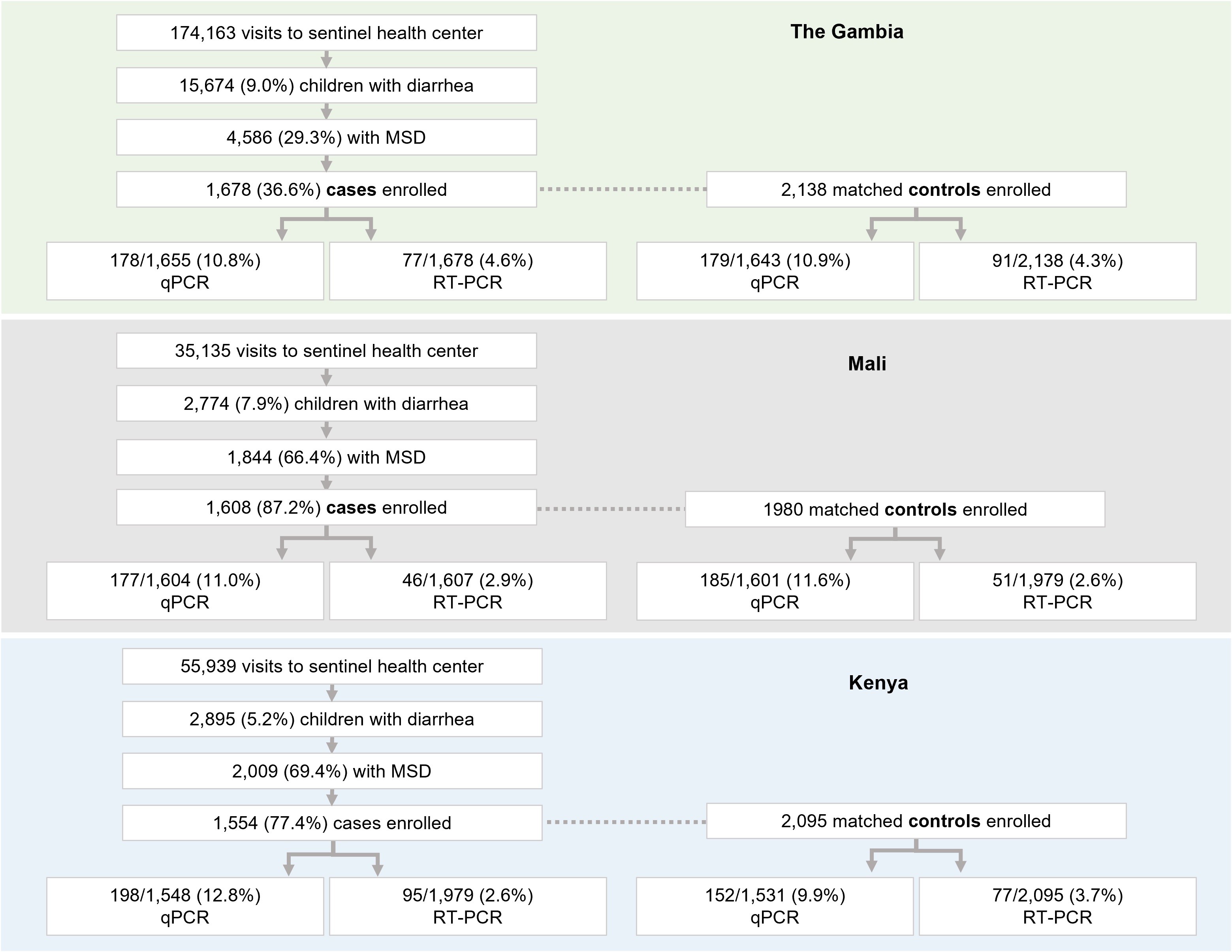

Supplement: ciac967_Supplementary_Data [file ciac967_supplementary_data.zip › Omore_Norovirus_SupFig1.tif]

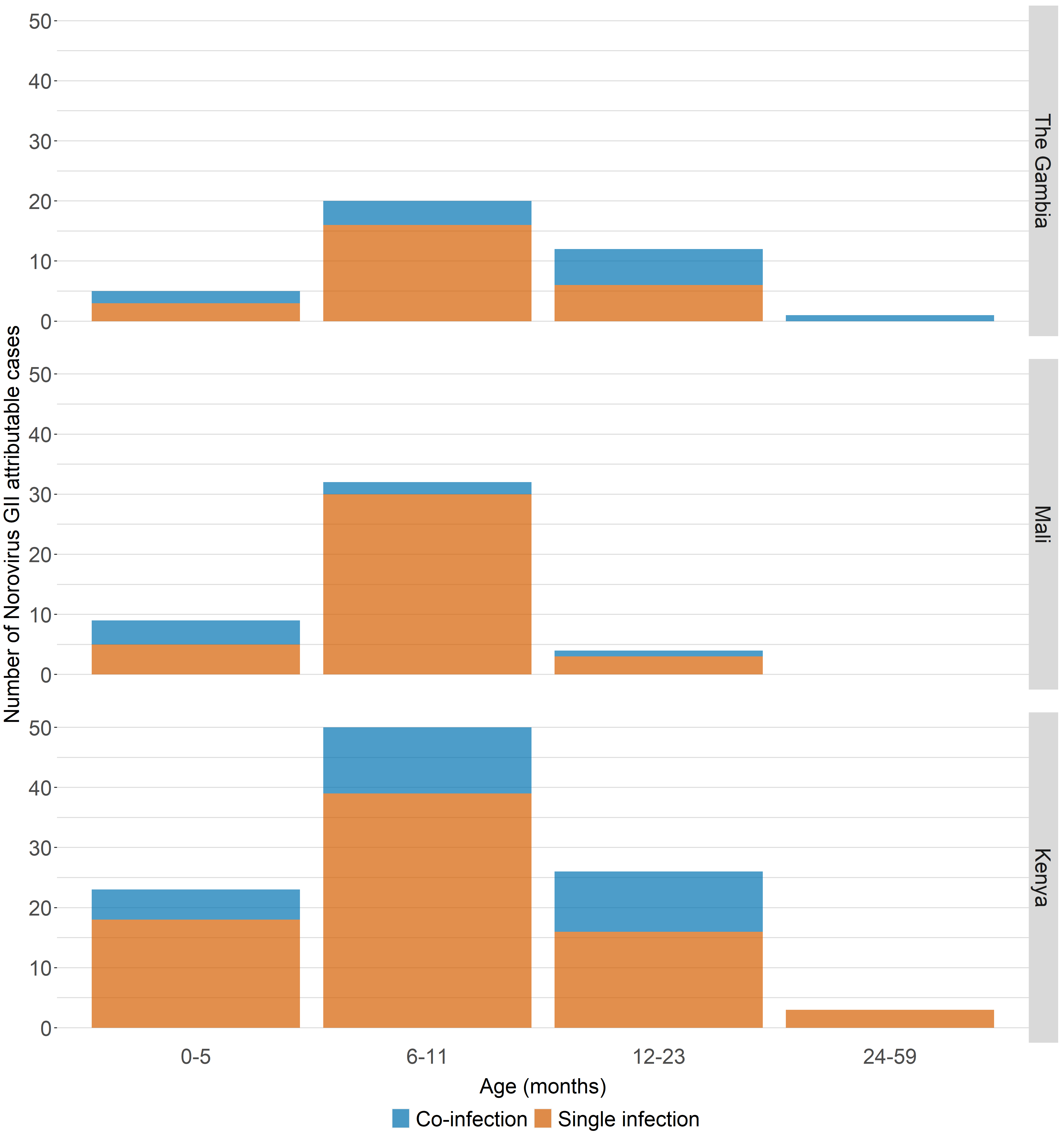

Supplement: ciac967_Supplementary_Data [file ciac967_supplementary_data.zip › Supp Fig 2a.tiff]

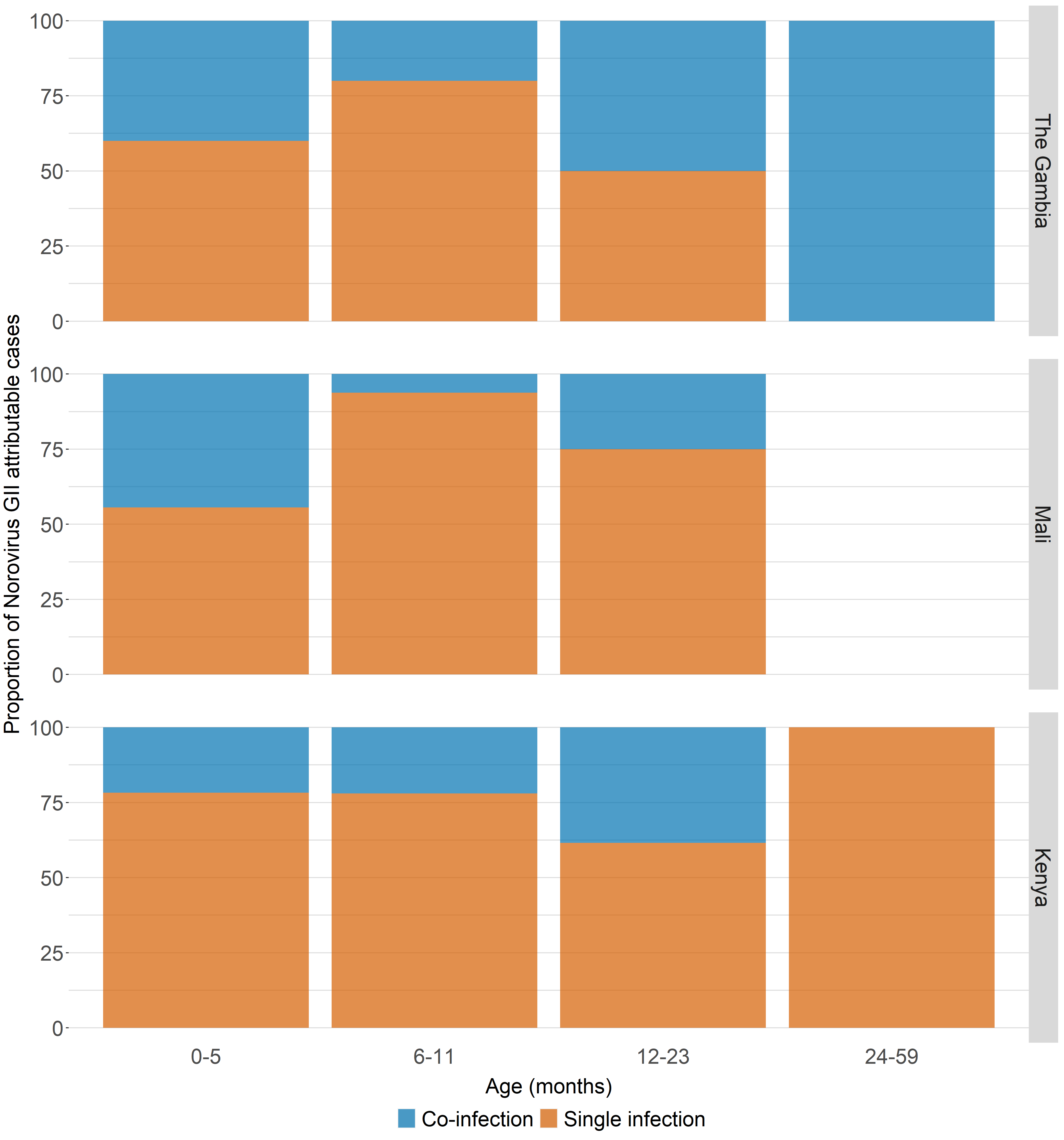

Supplement: ciac967_Supplementary_Data [file ciac967_supplementary_data.zip › Supp Fig 2b.tiff]
